# Development and characterization of rodent cardiac phantoms: comparison with in vivo cardiac imaging

**DOI:** 10.1016/j.mri.2012.04.008

**Published:** 2012-10

**Authors:** Steven Fortune, Maurits A. Jansen, Tom Anderson, Gillian A. Gray, Jürgen E. Schneider, Peter R. Hoskins, Ian Marshall

**Affiliations:** a Medical Physics and Medical Engineering, University of Edinburgh; b University and British Heart Foundation Centre for Cardiovascular Science, University of Edinburgh; c British Heart Foundation Experimental MR Unit, Department of Cardiovascular Medicine, University of Oxford

**Keywords:** Cardiac phantom, PVA cryogel, Gating, Cardiac MRI, Mouse, Rat

## Abstract

The increasing availability of rodent models of human cardiovascular disease has led to a need to translate noninvasive imaging techniques such as magnetic resonance imaging (MRI) from the clinic to the animal laboratory. The aim of this study was to develop phantoms simulating the short-axis view of left ventricular motion of rats and mice, thus reducing the need for live animals in the development of MRI.

Cylindrical phantoms were moulded from polyvinyl alcohol (PVA) Cryogel and attached via stiff water-filled tubing to a gear pump. Pulsatile distension of the phantoms was effected by suitable programming of the pump. Cine MRI scanning was carried out at 7 T and compared with in vivo rodent cardiac imaging.

Suitable pulsatile performance was achieved with phantoms for which the PVA material had been subjected to two freeze–thaw cycles, resulting in T1 and T2 relaxation time constants of 1656±124 ms and 55±10 ms, respectively. For the rat phantom operating at 240 beats per min (bpm), the dynamic range of the outer diameter was from 10.3 to 12.4 mm with the wall thickness varying between 1.9 and 1.2 mm. Corresponding figures for the mouse phantom at 480 bpm were outer diameter range from 5.4 to 6.4 mm and wall thickness from 1.5 to 1.2 mm.

Dynamic cardiac phantoms simulating rodent left ventricular motion in the short-axis view were successfully developed and compared with in vivo imaging. The phantoms can be used for future development work with reduced need of live animals.

## 1 Introduction

Time-resolved magnetic resonance imaging (MRI) of cardiac structure has become commonplace in human studies, and protocols are available from scanner manufacturers for use in clinical practice. Protocols typically include multiframe gradient-echo or steady-state free precession “cine” scans in standardized cardiac planes from which indices such as left ventricular (LV) volume, LV mass and ejection fraction can be evaluated. In recent years, the availability of rodent models of human disease has led to an increase in in vivo imaging studies of mice and rats. Small-animal MRI is at a less mature stage than human MRI, and recent effort has been concerned with the translation of imaging techniques from clinical systems to high-field, small-animal systems [1,2].

Phantoms are test devices which mimic some aspect of the behavior of tissues within the body and are used to provide test data sets for the purposes of development of new imaging techniques and for validation of measurements without need of human volunteers or experimental animals. In cardiac imaging, compensation of cardiac (and respiratory) motion, visualization of cardiac chamber motion and quantification of chamber volume are of interest. Human studies have used numerical phantoms [3–5] and static phantoms [6]. Dynamic phantoms have involved change in the volume of a chamber where measurement of the cardiac chamber volume is of interest [7–9] or change in the shape of a block of material such as polyvinyl alcohol (PVA) Cryogel where measurement of the strain in the myocardium is of interest [10,11]. Commercial cardiac phantoms suitable for MRI include a moving heart phantom (Limbs and Things, Bristol, UK) and a dynamic LV phantom (Shelley Medical Imaging, London, Canada). Very few phantoms for rodent cardiac MRI have been published. Li et al. [12] developed a static doughnut-shaped digital phantom for their work on myocardium imaging. Riegler et al. [13] described a phantom consisting of a heart extracted from a rat within which was inserted a balloon that was inflated to different volumes for calibration. Extending this to cyclic inflation would produce very realistic MRI data but with the disadvantage of requiring sacrifice of an animal, having a limited lifespan, involving biological tissues and not being easily reproducible by other labs. To date, there appears to be no reported work describing the design of a rodent phantom manufactured from readily available materials and not involving excised tissues or ex vivo preparations.

The aim of this work was to close this gap by developing cardiac phantoms suitable for rodent MRI. The phantoms were designed to provide realistic MRI data sets mimicking LV geometry and motion in the short-axis view.

## 2 Methods

### 2.1 Design criteria and design principles

The main criterion was to mimic the dynamic behavior of the heart in the short-axis (“cross-sectional”) view of the left ventricle at midventricular level. The phantoms should produce plausible MRI images, be of the same general dimensions as mouse and rat left ventricles, and undergo similar distension and change in wall thickness.

It was not the intention to model complex rotation and shortening movements or to mimic ventricular blood flow patterns. Previous studies have used a number of materials to construct cardiac phantoms, including agarose [6], latex [14], silicone [7,11,15] and PVA Cryogel [10]. The latter material is a gel, which has been used in the construction of ultrasound and MRI-compatible phantoms [16–19]. The gel is converted into an elastic solid by undergoing a number of freeze–thaw cycles. The elastic modulus and relaxation times T1 and T2 are controlled by the number of cycles, typically ranging between 2 and 10. PVA Cryogel was chosen for construction of the cardiac phantoms in this study because of the ability to readily control the characteristics of the material.

For cyclic distension of the phantom, two approaches were considered, namely, local activation and remote activation. Remote activation has been used in previous studies involving connection of the chamber to a remote pump via stiff tubing [9,11]. The potential disadvantage of this technique is loss of pulsatility due to some unavoidable elasticity of the tubing. Local activation could involve generation of a force close to the phantom. Possibilities might include use of the scanner's B_0_ magnetic field itself [20]. Though local activation would have been a technically elegant solution, a remote method was chosen as it is simple to implement and has worked in previous published studies.

### 2.2 Phantom construction

Phantoms were made to resemble the LV geometries of both rat and mouse. The dimensions of the phantoms were based on measurements made on healthy adult rats and mice in our laboratory. For both phantoms, an elongated hollow cylinder with a round end was manufactured. The mould consisted of an outer cylinder (a test tube) within which was centrally placed a Perspex rod. For the rat phantom, the outer diameter and wall thickness were nominally 10 mm and 2 mm, respectively, and for the mouse phantom, they were 5 mm and 1.5 mm, respectively. The central rod was raised above the bottom of the outer tube by an amount equal to the required wall thickness. A 15% concentration of PVA (PVA Gels, Kingston, NY, USA) in water was used. The PVA gel was heated to 80°C–95°C in a water bath, drawn into a 10-ml syringe and then injected into the mould to a depth of 2 cm for the rat phantom and 8 mm for the mouse phantom. The gel was allowed to settle overnight to allow any air bubbles to dissipate. The moulds were subject to two, four or six freeze–thaw cycles. Each cycle consisted of cooling at 0.5°C per minute to − 20°C, maintaining the temperature for 8 h and then allowing a rise to room temperature (22°C) at a rate of 0.5°C/min. The mould was maintained at room temperature for at least 8 h prior to separation of the PVA from the mould. The finished phantoms were stored in deionized water to prevent dehydration.

### 2.3 Phantom characterization

Relaxation time constants T1 and T2 have been reported for PVA at field strengths between 1 T and 3 T [16,17,21], but there are no reported values taken at higher magnetic field strengths. Phantoms were moulded from PVA; subjected to two, four and six freeze–thaw cycles; and then imaged in a 7-T MRI scanner [Agilent Technologies (formerly Varian, Inc.), Santa Clara, CA, USA]. Values of T1 were measured in the “short-axis” view using a fast spin echo sequence with inversion preparation and inversion times TI ranging from 10 ms to 3000 ms. The resulting image intensities were fitted to an exponential recovery curve using software on the scanner. Values of T2 were measured using fast spin echo sequences with echo times TE ranging from 10 ms to 60 ms, and the image intensities were fitted to an exponential decay curve using scanner software.

### 2.4 Phantom mounting and movement

**Fig. 1:**
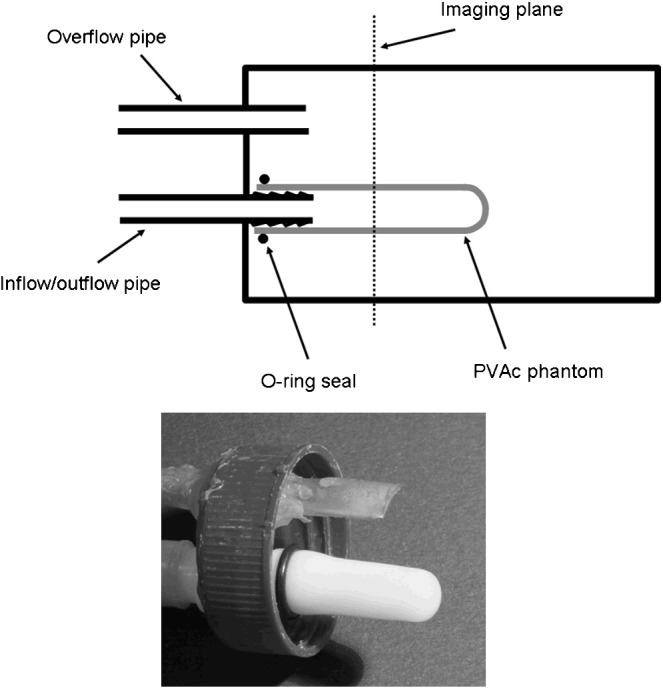
Rodent heart phantom. Upper panel shows the design of rodent cardiac phantoms based on polyvinyl alcohol Cryogel (PVAc) material moulded into a cylindrical shape with rounded end. The design outer diameter was 10 mm for rat and 5 mm for mouse phantoms. The enclosing chamber allows operation of the phantom in surrounding water. The lower panel shows a photograph of an example rat cardiac phantom.

The cardiac phantoms were mounted as shown in Fig. 1 within a sealed unit that could be filled with water and including an overflow as a precautionary measure in case of leakage during MRI scanning. The phantom was connected via stiff ¼-in. PTFE tubing (Cole-Parmer, Vernon Hills, IL, USA) to a gear pump (Michael Smith Engineering, Woking, UK). The phantom, tubing and gear pump were primed with water. The pump flow rate was controlled using a waveform generator. An offset sinusoidal waveform was applied in order to generate sinusoidal flow and hence cyclic distension of the phantom. Pumping frequencies up to 5 Hz [i.e., 300 beats per min (bpm)] were used for the rat phantom and up to 8 Hz (480 bpm) for the mouse phantom. Stroke volume and ejection fraction were varied by changing the waveform amplitude and offset.

### 2.5 MR dynamic imaging

The rat cardiac phantom assembly was placed in the 7-T scanner equipped with a 400-mT/m gradient set, and imaged with a 72-mm ID quadrature radiofrequency coil for transmission and a four-channel phased array coil for signal reception. Scout images enabled prescription of subsequent cine gradient-echo scanning using the manufacturer's standard sequence. One or more image slices were placed in the “short-axis” plane of the phantom. Image parameters were as follows: field of view (FOV)=42 mm, matrix 128×128, slice thickness 1.5 mm, four averages and minimum echo time. Repetition time was 10 ms, and flip angle was 20°. Trigger pulses from the pump controller were used to synchronize scanning with the motion of the phantom, and the number of time frames was adjusted to fit into the period of the motion. The mouse cardiac phantom was imaged with a 39-mm ID quadrature-driven transmit/receive coil and a 1000-mT/m gradient set. Other imaging parameters were as follows: FOV=30 mm, matrix 192×192, slice thickness 1 mm, three averages, repetition time 9.5 ms and flip angle 20°. Cine cardiac images were also acquired from anesthetized, healthy adult rats (Sprague–Dawley, bred in-house) and mice (C57Bl/6, bred in-house) using the same imaging parameters. All animal scanning complied with UK Home Office and University of Edinburgh regulations. Cardiac dimensions were measured from each time frame of the phantom image data sets and from the midventricular slice of representative rat and mouse image data sets using ImageJ software (http://rsbweb.nih.gov/ij). Outer and inner myocardial borders were fitted using elliptical contours, and radially averaged diameters and wall thicknesses were determined.

## 3 Results

### 3.1 PVA Cryogel T1 and T2 values

Values of T1 measured at 7 T were 1656±124, 1411±134 and 1334±96 ms for two, four and six freeze–thaw cycles, respectively, these figures being the mean±standard deviation over the imaged slices. The corresponding values for T2 were 55±10, 48±8 and 40±6 ms.

### 3.2 Phantom performance

**Fig. 2:**
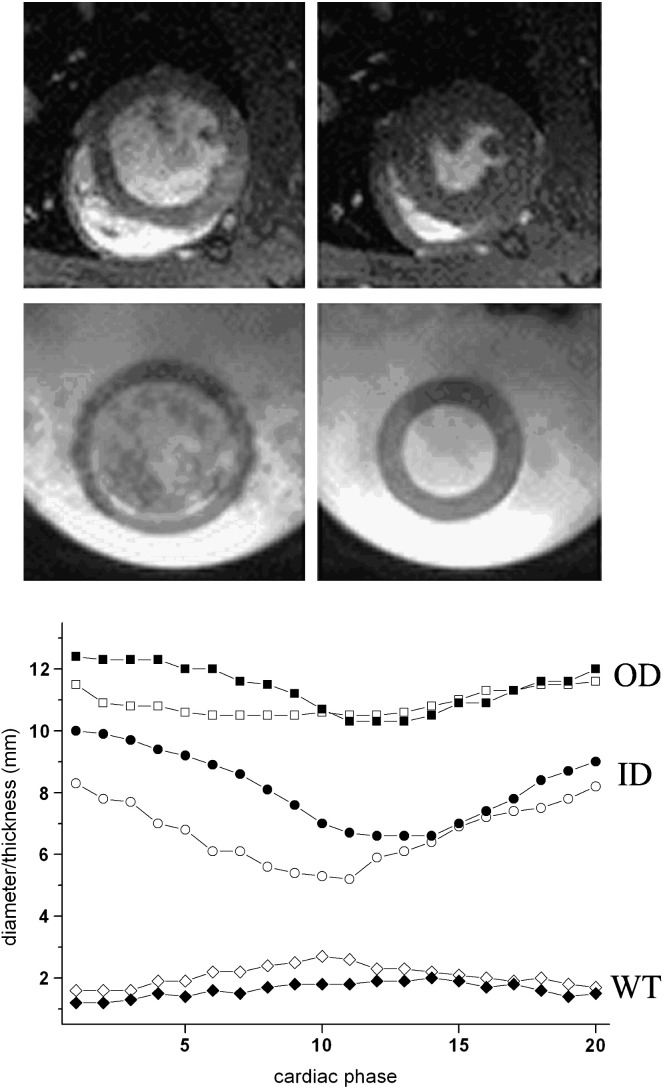
Rat cardiac imaging. Time frames from cine gradient-echo imaging of (upper row) an in vivo rat heart and (middle row) the rat cardiac phantom surrounded by water and operating at 240 bpm. Images correspond to (left) end diastole and (right) end systole. All images cropped to 20-mm square. Lower panel shows time course of LV wall motion. OD=outer diameter; ID=inner diameter, WT=wall thickness. Filled symbols, phantom; open symbols, in vivo. All measurements are radially averaged values. See also Table 1.

**Fig. 3:**
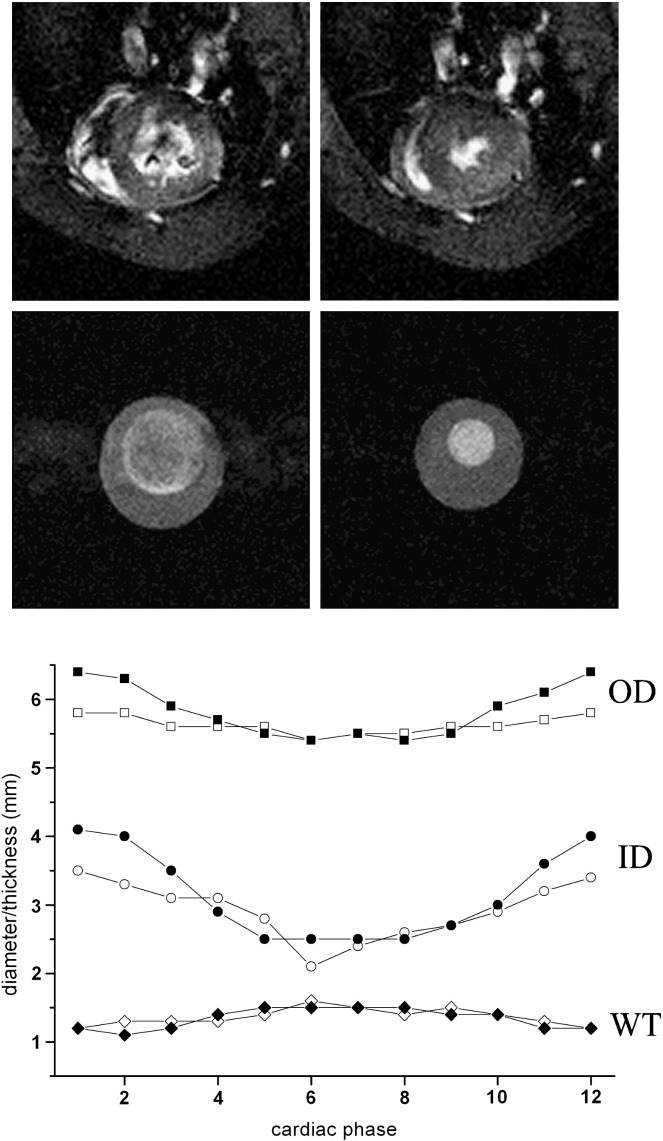
Mouse cardiac imaging. Time frames from cine gradient-echo imaging of (upper row) an in vivo mouse heart and (middle row) the mouse cardiac phantom in air, operating at 480 bpm. Images correspond to (left) end diastole and (right) end systole. All images cropped to 15-mm square. Lower panel shows time course of LV wall motion. Filled symbols, phantom; open symbols, in vivo. All measurements are radially averaged values. See also Table 1.

Preliminary experiments showed that phantoms made with two freeze–thaw cycles gave suitable distension with the pump system and were selected for the remainder of the study. Fig. 2 shows images of an in vivo rat heart compared with the rat cardiac phantom at end diastole and end systole when operating at 240 bpm. The cyclic changes in “left ventricular” diameter and wall thickness of the phantom are comparable with those of the live rat. Summary details of phantom and representative in vivo dimensions are given in Table 1 for both rat and mouse. Fig. 3 shows images of an in vivo mouse heart compared with the mouse cardiac phantom operating at 480 bpm, together with the corresponding time course of ventricular wall measurements.

## 4 Discussion

Left ventricular phantoms created using two freeze–thaw cycles of PVA material gave satisfactory performance compared with in vivo imaging of rats and mice.

We believe that these are the highest field measurements to date of the T1 and T2 properties of PVA Cryogel. Available literature values for T1 are approximately 400 ms, 600 ms, 800 ms and 1100 ms at 1 T, 1.5 T, 1.9 T and 3 T, respectively [16,21]. Our value of T1 of 1656 ms measured at 7 T confirms the overall trend of increasing T1 with field strength. For T2, there appears to be little change with field strength. The observed fall in T1 and T2 with the number of freeze–thaw cycles also confirms previous reports [16,17], although only the T1 values for two and four cycles reached statistical significance. Available literature values for myocardial T1 are 1300 ms in rat at 4.7 T [22] and 952 ms in mouse at 9.4 T [23], rather lower than our PVA Cryogel phantoms. However, our primary design goal was to generate realistic myocardial motion rather than exact matching of relaxation times.

Use of a pure sinusoidal flow from the pump resulted in eventual collapse of the phantom at “end systole,” so that an offset sinusoid was used. In practice, the amplitude and degree of offset were adjusted until the phantom operated without collapse. The use of an offset sinusoid would seem to imply an overall net flow towards the phantom. However, since no leaks were evident downstream of the pump, we conclude that the pump itself was not 100% efficient and that there was some backflow through it.

The phantoms exhibited smooth cyclic behavior with suitable pump settings, and the walls were highly visible in the MR images. As can be seen from Figs. 2 and 3 and Table 1, the dynamic range of diameters achieved was broadly similar to in vivo measurements except that the rat phantom had a larger inner diameter (and hence thinner walls) than a real rat heart (Fig. 2). Thin walls were necessary to ensure sufficient distensibility. The dynamic performance of the mouse phantom dimensions agreed very well with in vivo behavior, although some asymmetry of wall thickness is apparent in Fig. 3.

A limitation of the current phantoms is that their geometry is very simplified compared with real rodent hearts, but it is sufficient for imaging in the short-axis view routinely used in assessment of cardiac function [24]. Modeling of complex rotation and shortening movements was beyond the scope of the current work. The pattern of fluid flow within the phantom is quite different from blood flow in real hearts, but in this work, the objective was to mimic LV dimensions and not blood flow. Specifically, the phantoms were subsequently used to implement and test the kt-Broad-use Linear Acquisition Speed-up Technique [25] for accelerated cardiac imaging (data not shown).

Refinements beyond the scope of the current work could include the addition of rotation and “respiratory” motions, the incorporation of metabolites in the phantom walls for the development of MR spectroscopic techniques, and the use of a fully programmable pump to enable asymmetric timing of the cardiac cycle.

## 5 Conclusions

Dynamic cardiac phantoms simulating rodent LV short-axis motion were successfully developed and compared with in vivo imaging. The phantoms can be used for scanner testing and method development with reduced use of live animals.

## Figures and Tables

**Table 1:** Performance of rodent cardiac phantoms

	Rat (ED)	Rat (ES)	Mouse (ED)	Mouse (ES)
Phantoms				
Outer diameter	12.4	10.3	6.4	5.4
Inner diameter	10.0	6.6	4.0	2.2
Wall thickness	1.2	1.9	1.2	1.5
In vivo				
Outer diameter	11.5	10.5	5.8	5.4
Inner diameter	8.3	5.2	3.5	2.1
Wall thickness	1.6	2.6	1.2	1.6

Measurements made on rat and mouse LV cardiac phantoms compared with values from in vivo imaging. Rat phantom operating at 240 bpm and mouse at 480 bpm. ED=end diastole; ES=end systole. All dimensions are radially averaged values in mm. See also Figs. 2 and 3.
